# Red blood cell transfusion and outcomes in patients with acute lung injury, sepsis and shock

**DOI:** 10.1186/cc10458

**Published:** 2011-09-21

**Authors:** Elizabeth C Parsons, Catherine L Hough, Christopher W Seymour, Colin R Cooke, Gordon D Rubenfeld, Timothy R Watkins

**Affiliations:** 1 Division of Pulmonary and Critical Care Medicine, University of Washington, Harborview Medical Center, 325 Ninth Avenue, Seattle, WA 98104, USA; 2 Departments of Critical Care and Emergency Medicine, University of Pittsburgh, 3550 Terrace Street, Pittsburgh, PA 15261, USA; 3 Division of Pulmonary and Critical Care Medicine and Robert Wood Johnson Foundation Clinical Scholars Program, University of Michigan, 1150 West Medical Center Drive, Ann Arbor, MI 48109, USA; 4 Program of Trauma, Critical Care and Emergency Medicine, Sunnybrook Health Sciences Centre, University of Toronto, 2075 Bayview Avenue, Toronto, ON, M4N 3M5, Canada; 5 Research Institute, Puget Sound Blood Center, 921 Terry Avenue, Seattle, WA 98104, USA

**Keywords:** erythrocyte transfusion, respiratory distress syndrome, adult therapy, sepsis therapy, treatment outcome, intensive care unit, respiration, artificial

## Abstract

**Introduction:**

In this study, we sought to determine the association between red blood cell (RBC) transfusion and outcomes in patients with acute lung injury (ALI), sepsis and shock.

**Methods:**

We performed a secondary analysis of new-onset ALI patients enrolled in the Acute Respiratory Distress Syndrome Network Fluid and Catheter Treatment Trial (2000 to 2005) who had a documented ALI risk factor of sepsis or pneumonia and met shock criteria (mean arterial pressure (MAP) < 60 mmHg or vasopressor use) within 24 hours of randomization. Using multivariable logistic regression, we examined the association between RBC transfusion and 28-day mortality after adjustment for age, sex, race, randomization arm and Acute Physiology and Chronic Health Evaluation III score. Secondary end points included 90-day mortality and ventilator-free days (VFDs). Finally, we examined these end points among the subset of subjects meeting prespecified transfusion criteria defined by five simultaneous indicators: hemoglobin < 10.2 g/dL, central or mixed venous oxygen saturation < 70%, central venous pressure ≥ 8 mmHg, MAP ≥ 65 mmHg, and vasopressor use.

**Results:**

We identified 285 subjects with ALI, sepsis, shock and transfusion data. Of these, 85 also met the above prespecified transfusion criteria. Fifty-three (19%) of the two hundred eighty-five subjects with shock and twenty (24%) of the subset meeting the transfusion criteria received RBC transfusion within twenty-four hours of randomization. We found no independent association between RBC transfusion and 28-day mortality (odds ratio = 1.49, 95% CI (95% confidence interval) = 0.77 to 2.90; *P *= 0.23) or VFDs (mean difference = -0.35, 95% CI = -4.03 to 3.32; *P *= 0.85). Likewise, 90-day mortality and VFDs did not differ by transfusion status. Among the subset of patients meeting the transfusion criteria, we found no independent association between transfusion and mortality or VFDs.

**Conclusions:**

In patients with new-onset ALI, sepsis and shock, we found no independent association between RBC transfusion and mortality or VFDs. The physiological criteria did not identify patients more likely to be transfused or to benefit from transfusion.

## Introduction

Red blood cell (RBC) transfusion is common in the ICU, with nearly half of all critically ill patients receiving at least one transfusion during their ICU stay [1]. However, it is not clear that RBC transfusion improves patient outcomes. The use of RBC transfusion varies widely among physicians, with high rates of potentially unnecessary transfusions [1]. Several lines of evidence indicate that routine RBC transfusion in critically ill patients is associated with excess harm, including the development of nosocomial infection [2,3], acute lung injury (ALI) [4,5] and death [3,6-8].

Despite evidence linking RBC transfusion to adverse clinical outcomes and recommendations for lower transfusion thresholds, certain critically ill patients may benefit from RBC transfusion. RBC transfusions might benefit patients with sepsis by improving oxygen delivery while patients are in a state of high metabolic demand and overall oxygen deficit. A randomized, controlled trial supported this notion by demonstrating that an early goal-directed resuscitation protocol, including fluids, inotropes and RBC transfusion (at a hematocrit threshold of < 30%) saved lives when administered within 6 hours after severe sepsis diagnosis in the emergency department setting [9]. These results are in contrast to earlier studies of hemodynamically driven strategies aimed at supranormal oxygen delivery in the ICU, which failed to improve outcomes [10,11].

Conflicting evidence regarding RBC transfusion and outcomes has led to significant controversy over the use of RBC transfusion in goal-directed sepsis resuscitation strategies and in critically ill septic patients in the ICU [12,13]. A 2007 survey found that only 0.1% of responding physicians complied with all 2004 Surviving Sepsis Campaign guidelines advocating use of a goal-directed sepsis bundle that included RBC transfusion along with other therapeutics within the first 6 hours of resuscitation [14]. In this survey, protocol-driven RBC transfusion varied from 15% to 70% [14]. Current practice guidelines [12,13] do not address the use of RBC transfusion beyond the first 6 hours after sepsis diagnosis, despite evidence that in 43% of patients, the objectives of goal-directed therapy may not be initiated or completed within this time interval [15]. Furthermore, the effect of RBC transfusion on clinical outcomes in ICU patients with septic shock complicated by coexistent ALI is unknown. The Fluid and Catheter Treatment Trial (FACTT) trial showed that liberal volume administration (which could include RBC transfusion) was associated with poor outcomes in hemodynamically stable ALI patients [16], but the primary analysis did not examine the specific association between transfusion and clinical outcomes. In this study, we examined whether RBC transfusion administered in the ICU to patients with a recent diagnosis of ALI, sepsis and shock is independently associated with death and/or the number of days free from mechanical ventilation. We also investigated whether a prespecified set of physiological criteria might help identify a subset of patients most likely to receive or benefit from transfusion.

## Materials and methods

We performed a secondary analysis of the Acute Respiratory Distress Syndrome Network (ARDSNet) FACTT, a multicenter, randomized, controlled trial comparing the effectiveness of two fluid management and invasive monitoring strategies [16,17] performed between 2000 and 2005. FACTT enrolled 1,000 subjects within 48 hours of a new ALI diagnosis (mean time 24 hours at a median of 48 hours after hospital admission). All subjects were randomized to a liberal or conservative fluid management strategy and a pulmonary artery catheter or central venous catheter for 7 days or until they achieved unassisted ventilation. During periods of shock (defined as mean arterial pressure (MAP) < 60 mmHg or vasopressor use), fluid management was not dictated by the study protocol and left to the discretion of the clinician. Transfusion was not a part of the FACTT protocol and was initiated according to the physician's discretion. During the primary FACTT study, written informed consent was obtained from participants or legally authorized surrogates. All required data elements for our secondary analysis were available in their entirety from the FACTT database, acquired with permission from ARDSNet and the National Heart, Lung, and Blood Institute (NHLBI). The Institutional Review Board of the University of Washington approved this secondary data analysis and waived the need for additional consent.

### Eligibility and definitions

**Figure 1:**
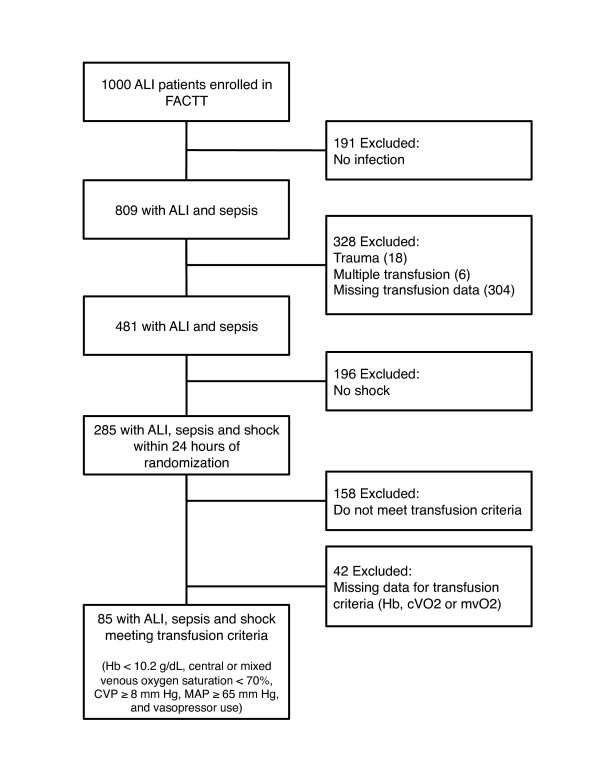
**Derivation of analysis cohorts**. ALI, acute lung injury; FACTT, Fluid and Catheter Treatment Trial; Hb, hemoglobin; cVO_2_, central venous oxygen saturation; mVO_2_, mixed venous oxygen saturation; CVP, central venous pressure; MAP, mean arterial pressure.

We first identified subjects with sepsis and shock within the FACTT database. We defined "sepsis" as the presence of a documented ALI risk factor for sepsis or pneumonia (Figure 1). We excluded subjects with a documented ALI risk factor for trauma or multiple transfusions, as well as those missing transfusion data during the first 24 hours after randomization. We defined "sepsis and shock" (hereinafter referred to as "shock") as a mean arterial pressure (MAP) < 60 mmHg or vasopressor use within the first 24 hours after randomization. Finally, we identified a subgroup of subjects with shock meeting four physiological criteria that might identify those subjects most likely to benefit from RBC transfusion (Figure 1). These criteria, derived from a sepsis resuscitation trial [9], included (1) adequate volume and pressor support, defined as central venous pressure (CVP) ≥ 8 mmHg, MAP ≥ 65 mmHg and use of a vasopressor; (2) poor perfusion, defined as central venous oxygen saturation (cVO_2_) or mixed venous oxygen saturation (mVO_2_) < 70%; and (3) anemia, defined as hemoglobin (Hb) < 10.2 g/dL.

### Data collection

Trained research coordinators collected demographic and clinical data prospectively during the FACTT study. These data included center, randomization arm, age, sex, race, location, APACHE III score 24 hours prior to randomization, baseline comorbidities, ratio of oxygen pressure to fraction of inspired oxygen (PaO_2_/FiO_2 _ratio), Hb and ventilatory parameters, including static pressure and tidal volume. In addition, detailed hemodynamic information was abstracted, including vasopressor use, MAP, cVO_2_, mVO_2 _and volume of fluid and blood products.

### Exposure

Transfusion data recorded at 8:00 AM daily through study day 8 were the number of packed RBC units transfused during the preceding 24 hours. Our goal was to examine the association between RBC transfusion and outcomes in subjects with a new ALI diagnosis who also met the criteria for sepsis and shock. We therefore restricted our transfusion exposure window to the first 24 hours after study randomization (a maximum of 72 hours after ALI diagnosis).

### Outcomes

The primary outcome was the proportion of patients who died before hospital discharge and within 28 days after study enrollment (28-day mortality). Patients were monitored during follow-up for 90 days or until death or discharge to home with unassisted breathing. Secondary outcomes were 90-day mortality and number of ventilator-free days (VFDs) by days 28 and 90 as previously defined [18].

### Statistical analysis

We performed bivariate comparisons between subjects who did or did not receive RBC transfusions using *t*-tests with unequal variance or Wilcoxon rank-sum tests for continuous variables and χ^2 ^tests for categorical variables. To assess the independent association between RBC transfusion and mortality at 28 and 90 days, we performed multivariable logistic regression, adjusting for factors which we considered potentially related to both outcomes and the likelihood of transfusion, including subject age [1,19,20], sex [19,20], race [19,21], APACHE III score and FACTT randomization arm [16]. To examine the association between RBC transfusion and VFDs, we performed multivariable negative binomial regression adjusted for the same predetermined confounders by using graphical analysis of predicted to observed probabilities and likelihood ratio testing to demonstrate goodness of fit. We then performed marginal means estimation to determine the adjusted difference in mean VFDs by transfusion status to assess goodness of fit using a log-likelihood ratio test [22].

Because of the prevalence of missing data for transfusion, we performed multiple imputation by chained equations to account for missing data [23-25]. Additional details regarding our imputation methods are given in Additional files 1 and 2. We repeated our primary analysis in the imputed cohort using Rubin's rules to generate combined risk estimates across the imputed datasets [26]. All statistical analyses were performed using Stata 11.0 software (State Corp., College Station, TX, USA).

## Results

### Derivation of the analysis cohorts

Of the 1,000 subjects enrolled in FACTT, 809 (81%) had ALI and a documented risk factor of sepsis and/or pneumonia. We excluded 328 subjects (33%) with an ALI risk factor of trauma (18 subjects), multiple transfusion (6 subjects) or missing transfusion data (304 subjects). We identified 285 subjects who met our criteria for shock within the first 24 hours after randomization (Figure 1). Of these 285 subjects, 85 (30%) met all transfusion indicators outlined above.

### Baseline characteristics

Fifty-three (19%) of the two hundred eighty-five subjects with shock were transfused within twenty-four hours of randomization, which occurred at a median of 1 day (interquartile range (IQR) 1 to 2 days) after ICU admission and a median of 2 days (IQR 1 to 5 days) after hospital admission. Transfused and nontransfused subjects were similar in terms of age, sex, ICU location, comorbidities, PaO_2_/FiO_2 _ratio and randomization arm (Table 1). In bivariate comparisons, transfusion was associated with black race (28% vs. 17%; *P *= 0.03), higher APACHE III score (mean 118 vs. 103; *P *< 0.01), more fluid administration in the first 24 hours (mean 6.8 vs. 5.5 L; *P *= 0.01) and lower baseline Hb (mean 8.5 vs. 9.7 g/dL; *P *< 0.01).

**Table 1:** Subject characteristics in shock

Characteristics	Transfused (*n *= 53)	Not transfused (*n *= 232)	*P *value
Age, years	53 (17)	52 (16)	0.52
Males	30 (57)	123 (53)	0.22
Race			
White	35 (66)	149 (64)	0.03
Black	15 (28)	40 (17)	
Other	3 (6)	43 (19)	
Chronic comorbidities			
Diabetes	11 (21)	40 (18)	0.59
Hepatic failure	2 (4)	1 (0.4)	0.03
Alcohol use	2 (4)	26 (12)	0.13
Prior myocardial infarction	0 (0)	11 (5)	0.12
Congestive heart failure	0 (0)	8 (4)	0.18
Admission type			
Medical ICU	47 (89)	211 (91)	0.87
Surgical ICU	5 (9)	18 (8)	
Other	1 (2)	3 (1)	
Randomization			
Liberal fluid (vs. conservative)	25 (47)	114 (49)	0.80
Pulmonary artery (vs. central venous) catheter	28 (53)	125 (54)	0.89
APACHE III score	118 (27)	103 (2)	< 0.01
Days from ALI diagnosis to randomization	0 (0, 1)	0 (0, 1)	0.99
Days from hospital admission to randomization	2 (1, 7)	2 (1, 4)	0.18
Days from ICU admission to randomization	1 (1, 2)	1 (1, 2)	0.89
PaO_2_/FiO_2 _ratio at randomization	107 (63 to 150)	108 (73 to 154)	0.47
Physiological parameters during exposure window			
Hemoglobin nadir, g/dL	8.5 (1.4)	9.7 (1.4)	< 0.01
cVO_2_/mVO_2 _ratio nadir	67 (12)	67 (13)	0.76
MAP nadir, mmHg	62 (8)	63 (9)	0.47
Mean MAP, mmHg	71 (8)	73 (9)	0.19
Total fluid received, L	6.8 (4.4)	5.5 (3.2)	0.01
Multiple pressors	27 (51)	120 (52)	0.92

APACHE III, Acute Physiology and Chronic Health Evaluation III; ALI, acute lung injury; cVO_2_, central venous oxygen saturation (%); mVO_2_, mixed venous oxygen saturation (%); MAP, mean arterial pressure (mmHg). Estimates are reported as *n *(%), means (SD) or medians (IQR) as appropriate.

### Outcomes

Twenty-three transfused subjects (43%) died by day 28 compared with 70 nontransfused subjects (30%) (*P *= 0.06). By day 28, median VFDs were zero (IQR 0 to 19) in transfused subjects and 9 (IQR 0 to 19) in nontransfused subjects (*P *= 0.35). In multivariable regression analysis, we observed no independent association between transfusion and 28-day mortality (adjusted odds ratio (OR) = 1.49, 95% CI = 0.77 to 2.90; *P *= 0.23) or VFDs (adjusted mean difference = -0.35, 95% CI = -4.03 to 3.32, *P *= 0.85) (Table 2). Likewise, we observed no independent association between transfusion and 90-day mortality (adjusted OR = 1.55, 95% CI = 0.81 to 2.96; *P *= 0.19) or VFDs (adjusted difference = -10.1, 95% CI = -23.6 to 3.42; *P *= 0.14) (Table 2). These results were not appreciably changed after performing multiple imputation of missing data (Additional file 2).

**Table 2:** Outcomes with red blood cell transfusion among subjects with shock

Parameter	Adjusted estimate^a ^(95% CI)	*P *value
Odds ratio for death		
At 28 days	1.49 (0.77 to 2.90)	0.23
At 90 days	1.55 (0.81 to 2.96)	0.19
Difference in mean ventilator-free days		
Days 1 to 28	-0.35 (-4.03 to 3.32)	0.85
Days 1 to 90	-10.1 (-23.6 to 3.42)	0.14

^a^Adjusted for age, gender, race, Acute Physiology and Chronic Health Evaluation III score and randomization arm. 95% CI, 95% confidence interval. Data in center column are odds ratio or marginal means estimates (95% CI).

### Subset analysis among subjects meeting transfusion criteria

In the subset of subjects meeting our prespecified transfusion criteria, only 20 (24%) received RBC transfusions during the exposure period of interest. Bivariate analyses of subject characteristics by transfusion status are shown in Table 3. Within this subgroup, transfusion was associated with older age (mean age = 65 vs. 51 years; *P *< 0.01) male sex (65% vs. 38%; *P *= 0.04), greater APACHE III scores (median = 122 vs. 103; *P *= 0.02) and lower Hb (mean = 8.2 vs. 9.0 g/dL; *P *= 0.02). Death by day 28 occurred in 10 (50%) of the transfused subjects compared to 19 (29%) of the nontransfused subjects (*P *= 0.09). By day 28, median VFDs were zero (IQR 0 to 12.5) in transfused subjects and nine (IQR 0 to 19) in nontransfused subjects (*P *= 0.26). In multivariable regression analysis, after adjusting for our predetermined confounders, we observed no independent association between transfusion and 28-day mortality (adjusted OR = 2.23, 95% CI = 0.63 to 7.81; *P *= 0.21) or VFDs (adjusted difference = -1.34, 95% CI = -7.50 to 4.82; *P *= 0.67) (Table 4). Likewise, we observed no independent association between RBC transfusion and 90-day mortality (adjusted OR = 2.16, 95% CI, 0.66 to 7.01; *P *= 0.20) or VFDs (adjusted difference = -18.4, 95% CI = -43.6 to 6.76; *P *= 0.15) (Table 4). These results were not appreciably changed after performing multiple imputation of missing data (Additional file 2).

**Table 3:** Subject characteristics in shock meeting physiological criteria for transfusion

Characteristics	Transfused (*n *= 20)	Not transfused (*n *= 65)	*P *value
Age, years	65 (15)	51 (14)	< 0.01
Male	13 (65)	25 (38)	0.04
Race			
White	14 (70)	45 (69)	0.49
Black	5 (25)	11 (17)	
Other	1 (5)	9 (14)	
Chronic comorbidities			
Diabetes	5 (26)	14 (22)	0.71
Hepatic failure	1 (5)	0 (0)	0.07
Alcohol use	0 (0)	9 (14)	0.09
Prior myocardial infarction	0 (0)	3 (5)	0.39
Congestive heart failure	0 (0)	5 (8)	0.26
Admission type			
Medical	16 (80)	61 (94)	0.08
Surgical	4 (20)	4 (6)	
Other	0 (0)	0 (0)	
Randomization arm			
Liberal fluid (vs. conservative)	11 (55)	29 (45)	0.42
Pulmonary artery (vs. central venous) catheter	14 (70)	46 (71)	0.95
APACHE III score	122 (7)	103 (3)	0.02
Days from ALI diagnosis to randomization	1 (0 to 1)	1 (0 to 1)	0.96
Days from hospital admission to randomization	2 (1 to 11)	2 (1 to 4)	0.49
Days from ICU admission to randomization	1 (0.5 to 1.5)	1 (1 to 2)	0.20
PaO_2_/FiO_2 _ratio at randomization	107 (60 to 144)	89 (66 to 152)	0.85
Physiological parameters during exposure window			
Hemoglobin nadir, g/dL	8.2 (1.2)	9.0 (0.8)	0.02
cVO_2_/mVO_2 _ratio nadir	59 (1)	59 (10)	0.99
MAP nadir	62 (7)	62 (9)	0.80
Mean MAP	72 (8)	73 (7)	0.70
Multiple pressors	12 (60)	32 (49)	0.45
Total fluid received, L	5.4 (3.0)	5.0 (2.9)	0.54

APACHE III, Acute Physiology and Chronic Health Evaluation III; ALI, acute lung injury; PaO_2_/FiO_2_, ratio of arterial oxygen pressure to fraction of inspired oxygen; cVO_2_, central venous oxygen saturation (%); mVO_2_, mixed venous oxygen saturation (%); MAP, mean arterial pressure (mmHg). Estimates are reported as *n *(%), means (SD) or medians (IQR) as appropriate.

**Table 4:** Outcomes with red blood cell transfusion among subjects with shock meeting physiological criteria for transfusion^a^

Parameter	Adjusted estimate (95% CI)^b^	*P *value
Odds ratio for death		
At 28 days	2.23 (0.63 to 7.81)	0.21
At 90 days	2.16 (0.66 to 7.01)	0.20
Difference in mean ventilator-free days		
Days 1 to 28	-1.34 (-7.50 to 4.82)	0.67
Days 1 to 90	-18.4 (-43.6 to 6.76)	0.15

^a^Transfusion criteria were defined by five simultaneous indicators: hemoglobin < 10.2 g/dL, central or mixed venous oxygen saturation < 70%, central venous pressure ≥ 8 mmHg, MAP ≥ 65 mmHg and vasopressor use. ^b^Adjusted for age, gender, race, Acute Physiology and Chronic Health Evaluation III score and randomization arm. Data in center column are odds ratio or marginal means estimates (95% CI).

## Discussion

The purpose of our study was to determine whether RBC transfusion administered in the ICU is associated with outcomes among patients with a recent diagnosis of ALI, sepsis and shock. We found that RBC transfusion in this period occurred in approximately one in five patients. The proportion of patients receiving RBC transfusions was similar in the subgroup of patients meeting our specified transfusion criteria. After adjusting for predetermined confounders, we found no significant, independent association between RBC transfusion and mortality or VFDs. The 95% CIs surrounding our risk estimates argue that the lack of statistical significance should be interpreted cautiously, as our risk estimates included clinically relevant differences in the direction of both benefit and harm.

Our study failed to show benefit or harm when RBC transfusion was administered to patients with a new diagnosis of ALI, sepsis and shock. There are several potential explanations for this result. First, RBC transfusion in this study was administered to patients in the ICU up to 72 hours after they met the criteria for sepsis and ALI. The clinical setting and/or timing of RBC transfusion may in fact be important in determining its benefit or harm [5,9,27,28]. A single randomized trial published by Rivers *et al*. [9] showed a mortality benefit when RBC transfusion was administered to patients with severe sepsis in the emergency department as part of a larger goal-directed resuscitation strategy that included fluid and vasopressor support. This resuscitation protocol was administered to enrolled subjects in an emergency department setting within 6 hours after a sepsis diagnosis. Thereafter, subjects were admitted to an ICU and underwent care as determined by their physicians. Notably, 64% of subjects in the treatment arm of the Rivers *et al*. trial were exposed to RBC transfusion within the first 6 hours of therapy. In contrast, observational studies in the ICU have not consistently demonstrated that RBC transfusion improves oxygen delivery in fluid-replete septic subjects [28-30] and instead raises concern regarding increased complications, including nosocomial infection [2,3,31], ALI [3-5,8] and death [3,6-8,32]. In an observational study of 160 ICU patients with septic shock, delayed goal-directed resuscitation and transfusion up to 48 hours after diagnosis were associated with higher risk of ALI [5]. Similarly to these observational studies, our findings may reflect a lack of benefit when transfusion is administered beyond the initial 6-hour resuscitation window or for reasons other than protocol-driven resuscitation in severe sepsis. Finally, RBC transfusion may carry minimal beneficial effects or even harmful effects on patient outcomes independently of other resuscitative strategies, such as volume resuscitation or vasopressor support.

Despite our efforts to identify a subset of subjects with shock whom transfusion might benefit, we observed no improvement in outcomes with RBC transfusion. Although transfusion criteria were met in one of four subjects, we observed no treatment association when adjusting for these factors in subgroup analysis. Consistent with prior work [28-30,33], our study suggests that physiological indicators may not necessarily identify those patients likely to benefit from RBC transfusion. While randomized data in patients with septic shock are lacking, there is growing experimental evidence that transfusion of stored RBCs can potentially harm patients with preexisting inflammation or impaired microvascular perfusion. According to the current "two-hit" hypothesis of transfusion injury [34], RBC units may contain bioactive particles capable of influencing the cellular injury that leads to organ failure in susceptible patients with preexisting insults such as sepsis or mechanical ventilation [4,34,35]. In addition, *in vivo *models have demonstrated that older RBC units exhibit reduced deformability [36,37], which may actually impair capillary flow and oxygen delivery in an already compromised microvascular system [38,39]. It is therefore possible that RBC transfusion administered beyond the first 6 hours of illness may paradoxically be harmful in the very patients that we hope will benefit.

Our study has several limitations. First, transfusion data for a significant number of subjects were missing. Because complete case analysis in the setting of missing data may be limited by both reduced power and residual bias [23,26], we performed a sensitivity analysis using multiple imputation of missing values, which provided results similar to our primary analysis. The combination of missing RBC transfusion data and the small proportion of patients who met our "shock" definition limited our study's power to detect statistically significant associations between transfusion and outcomes (minimum detectable difference in mortality = 19% based on an overall mortality rate of 30% and two-sided α = 0.05). While pooled blood products such as fresh frozen plasma (FFP) may also have an effect on patient outcomes, missing data and low FFP transfusion rates in our cohort precluded our ability to include FFP as a meaningful covariate. Furthermore, we cannot exclude the possibility of residual bias related to unignorable missing data (missing not at random) or other covariates either not present or insufficiently captured in the database, including age of transfused blood [40], the indication for transfusion, concomitant therapies such as fluid administration and the manner in which transfusion was administered. Though we carefully defined sepsis, shock and physiological criteria on the basis of objective measures within a fixed time period, misclassification of shock due to etiologies other than sepsis is a potential limitation of our study. We also could not determine the reason why physicians chose to transfuse individuals or whether transfusion was administered concomitantly with other resuscitation strategies. The decision to administer RBC transfusion may depend on a host of factors, including patient-, hospital- and provider-level characteristics [1,14]. Understanding factors that contribute to transfusion practice variability is an important avenue of future study, because blood products are a limited and costly health care resource. Last, our study cannot determine whether RBC transfusion is a meaningful component of early goal-directed therapy within the first 6 hours of severe sepsis. It is important to note that our patients likely differed significantly, with regard to their stage of illness, indication for transfusion and concomitant therapy, from those enrolled in the randomized trial evaluating early goal-directed therapy in the emergency department setting [9]. Nonetheless, some form of goal-directed resuscitation likely extends beyond the first 6 hours of severe sepsis into the ICU period. Previous work suggests that delayed goal-directed therapy may be associated with increased complications in critically ill septic patients [5]. Despite its limitations, our study builds on previous work suggesting that RBC transfusion beyond 6 hours of presentation may not improve mortality in critically ill patients with septic shock and coexistent ALI and that physiological criteria may not identify those patients likely to benefit from transfusion in the ICU setting.

## Conclusions

We did not observe a statistically significant benefit or harm associated with RBC transfusion among patients with a recent diagnosis of ALI, sepsis and shock. In addition, there was no statistically significant difference in outcomes among the subset of subjects meeting prespecified physiological transfusion criteria. While not meeting statistical significance, our observed risk estimates do not exclude the possibility of clinically relevant transfusion-related benefit or harm. These data add to our understanding of the use of RBC transfusion in patients with a recent diagnosis of ALI undergoing resuscitation in the ICU, suggesting that physiological indicators may not identify those patients likely to benefit from transfusion therapy. Future studies are needed to verify these results in larger cohorts to account for potential modifiers, including age of transfused blood and other resuscitative strategies.

## Key messages

• RBC transfusion is of unclear benefit to patients with established ALI and severe sepsis.

• In this study, physiological criteria did not identify patients more likely to be transfused or to benefit from transfusion.

• Future studies are needed to examine potential modifiers, including age of transfused blood and other resuscitative strategies.

## Abbreviations

ALI: acute lung injury; APACHE III: Acute Physiology and Chronic Health Evaluation III; cVO_2_: central venous oxygen saturation; FACTT: Fluid and Catheter Treatment Trial; MAP: mean arterial pressure; mVO_2_: mixed venous oxygen saturation; RBC: red blood cell.

## Competing interests

The authors declare that they have no competing interests.

## Authors' contributions

EP participated in the study design, data acquisition, statistical analysis and data interpretation and also drafted the manuscript. CH participated in the statistical analysis, data interpretation and manuscript revision. CW and CC participated in the multiple imputation analysis and manuscript revision. GR participated in study conception and revised the manuscript critically for important intellectual content. TW participated in study conception, study design, statistical analysis, data interpretation and manuscript revision. All authors read and approved the final manuscript.

## Supplementary Material

Additional file 1 **Methods for Missing Data Analysis**. This file presents subject characteristics among patients with shock, according to the presence or absence of RBC transfusion data. It also details the methods used for multiple imputation of missing data for RBC transfusion and other covariates. **Table E1 Subject characteristics in shock by the presence or absence of transfusion data**. ALI, acute lung injury; APACHE III, Acute Physiology and Chronic Health Evaluation III; MICU, medical ICU; SICU, surgical ICU; PaO_2_/FiO_2_, ratio of arterial oxygen pressure to fraction of inspired oxygen; cVO_2_/mVO_2_, ratio of central venous oxygen saturation to mixed venous oxygen saturation; MAP, mean arterial pressure; SD, standard deviation; IQR, interquartile range. **Table E2 Imputation variables with number of missing values in subjects with shock**. ALI, acute lung injury; MICE, multiple imputation using chained equations; FACTT, Fluid and Catheter Treatment Trial; APACHE III, Acute Physiology and Chronic Health Evaluation III; MICU, medical ICU; SICU, surgical ICU; PaO_2_/FiO_2_, ratio of arterial oxygen pressure to fraction of inspired oxygen; PaO_2_, arterial oxygen pressure; PaCO_2_, arterial carbon dioxide pressure; BUN, blood urea nitrogen; MAP, mean arterial pressure; CVP, central venous pressure. Click here for file

Additional file 2 **Results of Imputation Analysis**. This file presents the results of multivariate regression analysis performed in the imputation cohort. **Table E3 Outcomes with red blood cell transfusion among subjects with sepsis and shock within the imputation cohort**. ALI, acute lung injury; 95% CI, 95% confidence interval; APACHE III, Acute Physiology and Chronic Health Evaluation III. Click here for file
