# Supplementary material for: Red blood cell transfusion and outcomes in patients with acute lung injury, sepsis and shock
Source: Crit Care. 2011 Sep 21;15(5):R221. doi: 10.1186/cc10458 (PMC3334766; doi:10.1186/cc10458)
Supplement: Additional file 1 — Methods for Missing Data Analysis. This file presents subject characteristics among patients with shock, according to the presence or absence of RBC transfusion data. It also details the methods used for multiple imputation of missing data for RBC transfusion and other covariates. Table E1 Subject characteristics in shock by the presence or absence of transfusion data. ALI, acute lung injury; APACHE III, Acute Physiology and Chronic Health Evaluation III; MICU, medical ICU; SICU, surgical ICU; PaO2/FiO2, ratio of arterial oxygen pressure to fraction of inspired oxygen; cVO2/mVO2, ratio of central venous oxygen saturation to mixed venous oxygen saturation; MAP, mean arterial pressure; SD, standard deviation; IQR, interquartile range. Table E2 Imputation variables with number of missing values in subjects with shock. ALI, acute lung injury; MICE, multiple imputation using chained equations; FACTT, Fluid and Catheter Treatment Trial; APACHE III, Acute Physiology and Chronic Health Evaluation III; MICU, medical ICU; SICU, surgical ICU; PaO2/FiO2, ratio of arterial oxygen pressure to fraction of inspired oxygen; PaO2, arterial oxygen pressure; PaCO2, arterial carbon dioxide pressure; BUN, blood urea nitrogen; MAP, mean arterial pressure; CVP, central venous pressure. [file cc10458-S1.DOC]

**Additional File 1.** Methods for Missing Data Analysis

*Subject Identification*

We first identified subjects with ALI, sepsis and shock who did and did not have transfusion data in the first 24 hours after randomization. We then examined subject characteristics by the presence or absence of transfusion data for the first 24 hours (Table E1). There were no major differences between subjects with and without transfusion data, with the exception that those with transfusion data were more likely to receive diuretics.

**Table E1: Subject characteristics in shock by the presence or absence of transfusion data**

|  | Transfusion Data Present  (Primary Analysis)  (N=285) | Transfusion Data  Absent  (N=167) | P value |
| --- | --- | --- | --- |
| Age, yr | 52 (16) | 52 (17) | 0.91 |
| Male | 153 (54) | 82 (49) | 0.35 |
| Race |  |  |  |
| White | 184 (65) | 104 (62) | 0.88 |
| Black | 55 (19) | 35 (21) |  |
| Other | 46 (16) | 28 (17) |  |
| Location |  |  |  |
| MICU | 217 (76) | 123 (74) | 0.37 |
| SICU | 10 (4) | 3 (1.8) |  |
| Other | 58 (20) | 41 (24.6) |  |
| APACHE III | 105 (82, 126) | 110 (89, 129) | 0.22 |
| Randomized to |  |  |  |
| Liberal fluid arm | 139 (49) | 81 (49) | 0.96 |
| Pulmonary artery catheter | 153 (54) | 91 (54) | 0.87 |
| PaO2/FiO2 at randomization | 108 (71, 154) | 97 (71, 150) | 0.29 |
| Physiologic indicators within the 1^st^ 24h of randomization |  |  |  |
| Hemoglobin Nadir, g/dL | 9.5 (1.5) | 9.6 (1.5) | 0.27 |
| cVO2/mVO2 Nadir, % | 67 (13) | 68 (12) | 0.50 |
| MAP Nadir | 63 (9) | 62 (10) | 0.84 |
| Mean MAP | 72 (9) | 71 (9) | 0.57 |
| Multiple vasopressors | 161 (56) | 96 (57) | 0.83 |
| Total fluids, liters | 5.8 (4) | 5.4 (3) | 0.24 |
| Diuretics administered | 92 (32) | 28 (18) | <0.01 |
| Baseline Comorbidities |  |  |  |
| Diabetes | 51 (19) | 37 (22) | 0.33 |
| Hepatic failure | 3 (1) | 2 (1) | 0.91 |
| Alcohol use | 28 (10) | 14 (9) | 0.80 |
| Prior myocardial infarction | 11 (4) | 7 (5) | 0.72 |
| Congestive heart failure | 8 (3) | 4 (3) | 0.90 |

Estimates reported as N(%), mean (sd), or med(IQR) as appropriate.

*Missing data procedures: Multiple Imputation*

Missing data was common in this dataset (Table E1). Transfusion data in the first 24 hours after randomization was missing in 38% of patients with ALI, 37% of patients with shock, and 31% of patients with shock meeting transfusion criteria. We assumed that missing data was “missing at random” and therefore used a flexible multivariable imputation procedure of multiple chained regression equations (multiple imputation by chained equations, i.e. MICE) which generated values for all missing data using the observed data for all patients. We included the model covariates (N=6), our 28-day outcomes (N=2), and auxiliary variables (N=23, Table E2) in the imputation procedure. Auxiliary variables were predetermined variables derived from the FACTT database.

We modeled 28-day and 90-day mortality using multinominal logistic regression. We log-transformed non-normally distributed variables (e.g. hospital length of stay) prior to imputation. MICE may still lead to bias in the setting of > 50% missing data, but this bias is generally less than that resulting from complete case analysis (which assumes missing completely at random). We used 10 switching cycles to create 40 independent datasets. We used predictive mean matching for continuous, non-normally distributed variables with upper and lower bounds (ventilator free days) .

**Table E2:** Imputation variables with number of missing values in subjects with shock (N=452)

| Variable | Missing Values  N (%) |
| --- | --- |
| Exposure |  |
| Transfusion during the 1^st^ 24 hours after randomization | 167 (37) |
| Outcomes |  |
| 28 day mortality | 0 (0) |
| 28 day ventilator free days | 0 (0) |
| Primary Model Covariates |  |
| Age | 0 (0) |
| Sex | 0 (0) |
| Race | 0 (0) |
| Randomization arm | 0 (0) |
| APACHE III score | 13 (2.8) |
| Additional Imputation Variables |  |
| Place of residence prior to hospitalization^a^ | 57 (12.6) |
| Location (MICU, SICU, other) | 0 (0) |
| Positive blood culture during hospitalization | 0 (0) |
| Admission type (medical, surgical, other) | 0 (0) |
| Hospital length of stay^b^ | 0 (0) |
| PaO2/FiO2 ratio day 0^a^ | 24 (5.3) |
| Vasopressors at randomization | 0 (0) |
| Baseline tidal volume^b^ | 46 (10.2) |
| Baseline plateau pressure | 132 (29.2) |
| Baseline PaO2^b^ | 15 (3.3) |
| Baseline PaCO2^b^ | 15 (3.3) |
| Baseline wbc count ^b^ | 2 (0.4) |
| Baseline platelet count ^b^ | 2 (0.4) |
| Baseline sodium | 1 (0.2) |
| Baseline potassium | 0 (0) |
| Baseline glucose ^b^ | 5 (1.1) |
| Baseline BUN ^b^ | 0 (0) |
| MAP nadir 1^st^ 24hr | 0 (0) |
| CVP nadir 1^st^ 24hr | 3 (0.7) |
| Hemoglobin nadir 1^st^ 24hr | 1 (0.7) |
| Fluid administered in the 1^st^ 24hr | 13 (2.8) |
| Baseline total protein | 50 (11.1) |
| Baseline bicarbonate | 2 (0.4) |

^a^ home, skilled care/rehabilitation facility, acute care, or other

^b^ transformed to logarithm

**REFERENCES**

1. Royston P: **Multiple imputation of missing values.** *Stata Journal* 2004, **4**:227-241.

2. van Buuren S: **Multiple imputation of discrete and continuous data by fully conditional specification**. S*tat Methods Med Res 2007*, **16**:219-242.

3. Landerman LR LK, Pieper CF: **An Empirical Evaluation of the Predictive Mean Matching Method for Imputing Missing Values.** *Sociological Methods Research* 1997, **26**:3-33.
