# Supplementary material for: Red blood cell transfusion and outcomes in patients with acute lung injury, sepsis and shock
Source: Crit Care. 2011 Sep 21;15(5):R221. doi: 10.1186/cc10458 (PMC3334766; doi:10.1186/cc10458)
Supplement: Additional file 2 — Results of Imputation Analysis. This file presents the results of multivariate regression analysis performed in the imputation cohort. Table E3 Outcomes with red blood cell transfusion among subjects with sepsis and shock within the imputation cohort. ALI, acute lung injury; 95% CI, 95% confidence interval; APACHE III, Acute Physiology and Chronic Health Evaluation III. [file cc10458-S2.DOC]

**Additional File 2.** Results of Imputation Analysis

In addition to the 285 subjects in the primary analysis, we identified 167 subjects who met criteria for shock but had missing transfusion data, yielding a total of 452 patients in the imputation cohort. Of these, 124 subjects met physiologic criteria for transfusion. We performed the same regression analysis in these subjects, yielding similar results to the primary analysis (Table E3).

**Table E3:** Outcomes with RBC transfusion among subjects with sepsis and shock within the Imputation Cohort

|  | ALI, sepsis and shock  (N=452) | | ALI, sepsis and shock meeting physiologic criteria for transfusion  (N=124) | |
| --- | --- | --- | --- | --- |
|  | Adjusted Estimate *^a^*  (95% CI) | P value | Adjusted Estimate *^a^*  (95% CI) | P value |
| Odds Ratio for Death |  |  |  |  |
| At 28 days | 1.37 (0.72, 2.60) | 0.34 | 1.50 (0.51, 4.36) | 0.46 |
| At 90 days | 1.36 (0.73, 2.52) | 0.33 | 1.27 (0.45, 3.59) | 0.65 |
| Difference in Mean Ventilator Free Days |  |  |  |  |
| Days 1 to 28 | -0.83 (-3.87, 2.20) | 0.64 | -0.22 (-4.46, 4.01) | 0.92 |
| Days 1 to 90 | -8.43 (-17.4, 0.58) | 0.25 | -4.76 (-17.8, 8.32) | 0.70 |

*^a^* Adjusted for age, gender, race, APACHE, randomization arm.
